# Development of two socioeconomic indices for Saudi Arabia

**DOI:** 10.1186/s12889-018-5723-z

**Published:** 2018-06-26

**Authors:** Reem S. AlOmar, Roger C. Parslow, Graham R. Law

**Affiliations:** 1 Department of Family and Community Medicine, Imam Abdulrahman Bin Faisal University, Dammam, Saudi Arabia; 2 Division of Epidemiology and Biostatistics, University of Leeds, Leeds, UK; 3 Community and Health Research Unit, School of Health and Social Care, University of Lincoln, Lincoln, UK

**Keywords:** Saudi Arabia, Socioeconomic status, Exploratory factor analysis, Latent class analysis, Deprivation

## Abstract

**Background:**

Health and socioeconomic status (SES) are linked in studies worldwide. Measures of SES exist for many countries, however not for Saudi Arabia (SA). We describe two indices of area-based SES for SA.

**Methods:**

Routine census data has been used to construct two indices of SES at the geographically-delimited administrative region of Governorates in SA (*n* = 118). The data used included indicators of educational status, employment status, car and material ownership. A continuous measure of SES was constructed using exploratory factor analysis (EFA) and a categorical measure of SES using latent class analysis (LCA). Both indices were mapped by Governorates.

**Results:**

The EFA identified three factors: The first explained 51.58% of the common variance within the interrelated factors, the second 15.14%, and the third 14.26%. These proportions were used in the formulation of the standard index. The scores were fixed to range from 100 for the affluent Governorate and 0 for the deprived. The LCA found a 4 class model as the best model fit. Class 1 was termed “affluent” and included 11.01% of Governorates, class 2 “upper middle class” (44.91%), class 3 “lower middle class” (33.05%) and class 4 “deprived” (11.01%). The populated urbanised Governorates were found to be the most affluent whereas the smaller rural Governorates were the most deprived.

**Conclusion:**

This is the first description of measures of SES in SA at a geographical level. Two measures have been successfully constructed and mapped. The maps show similar patterns suggesting validity. Both indices support the common perception of SES in SA.

**Electronic supplementary material:**

The online version of this article (10.1186/s12889-018-5723-z) contains supplementary material, which is available to authorized users.

## Background

Inequality in health outcomes, and access to health services and their utilisation due to socioeconomic status (SES) is a common theme in health research and policy intervention [1]. It is acknowledged that SES affects both morbidity and mortality, and has been linked to low birth weight, cardiovascular diseases, arthritis, hypertension, diabetes and cancer [2, 3].

The trend in health research has shifted from the sole use of individual-based socioeconomic data to an approach that includes area-based aggregate data, this approach is more convenient as one composite index can be easily included into public health surveillance programs, as opposed to adding several individual data items [4]. This analysis typically includes matching individuals’ residential information such as postcodes to a spatial location, thus creating area-profiles that are easy to use in research [5].

Focus on health disparities attributable to SES allows governments and health organisations to target interventions, especially in deprived populations [6, 7]. In the UK for example, the measurement of SES has a long history, where indices such as Townsend [8], Carstairs [9] and the IMD [10] have been developed and are continuously updated for health research and policy intervention purposes. Other countries have also developed their own area-based indices of SES, in Europe [11], the US [12], Canada [13] and others [14, 15].

Measurements of SES in the Gulf countries and in Saudi Arabia (SA) specifically, are not well developed. The standard measure of SES used in health research in these countries rely solely on individual characteristics such as income, and educational status and these measures fail to account for the social and health context [16, 17]. To our knowledge, no study has yet examined the relationship between any health outcome and SES on an area-based level in the Gulf region, because no area-based measure exists for any of these countries. In SA, the problem persists, but is worsened by the fact that SA is the largest country in the Gulf [18]. Unlike the UK and other industrialised countries, it is a common perception that rural areas in SA are the most deprived in terms of education, income and health outcomes, where travelling distances to higher educational institutions and specialised health organisations are larger for those living within the more rural areas.

Therefore, the present study aims to formulate two socioeconomic indices for SA at the Governorate level, and provide geographical representations of them. These indices will explore and possibly validate the common perception of SES in SA.

## Methods

### Population data

The national census data of 2004 from the General Authority for Statistics in SA was used for this analysis [19]. There are two main geographical levels for the country, the 13 provinces, and nested within these are 118 Governorates. Each province has a different number of Governorates. The average population within the Governorates is 192,189, and the median is 57,792. The population ranges from 4,138,329 in the capital Riyadh to 3785 in Kharkheer. The geographical level of analysis in this study is the Governorate level, since that is the geographical level at which most administrative and health data are collected, and also for data accessibility issues.

### Indicator variables

The national census of SA does not collect information on a specific measure of social class explicitly, and household income and overcrowding data was not disclosed by those surveyed [19]. The accessible indicators (available in aggregated form at: https://www.stats.gov.sa/en) used are summarised in Table 1.

**Table 1:** Indicators used in the measurement of socioeconomic status for Saudi Arabia

Categories	Indicator variables	Denominator
Educational status	Illiterate and read/write	Population aged > 10 years
	School degree	
	Diploma and university	
	Higher education	
Employment status^a^	In the labour force	Population aged > 15 years
	Students	
	Housewives	
	Retired	
	Other employment	
Type of housing	Traditional house	Households
	Villa	
	A floor in a traditional house or villa	
	Apartment	
	Other type of housing	
Tenure of housing^b^	Owned	Households
	Rented	
	Provided	
	Other tenure	
Car ownership	No car	Households
	One car	
	Two or more cars	
Material ownership	Phone available/not available	Households
	TV available/not available	
	PC available/not available	
	Internet available/not available	
	Library available/not available	
	Satellite available/not available	
	Videos available/not available	
	Video games available/not available	

^a^In the latent class analysis employment status the following indicators: “Students”, “Housewives” and “Retired” were combined to “Not in the labour force”

^b^In the latent class analysis tenure of housing indicators the following indicators: “Rented”, “Provided” and “Other tenure” were combined into “House not owned”

### Digital boundary data

Digital boundaries of the 118 Governorates of the country facilitated geo-referenced data linkage and were provided from Farsi GeoTech Company [20].

#### Statistical analysis

Two indices of SES will be developed using two different statistical methodologies. The motive is to come up with two types of indices, one continuous with detailed information on each Governorate (Standardised index of SES), and the other categorical where several similar Governorates are combined into one category or class (Socioeconomic classes).

### Standardised index of SES

An exploratory factor analysis (EFA) was used to estimate the underlying latent structure of the indicators. The assumption underlying EFA is that the correlations between the indicators are from the common factors [21].

First, all indicators were expressed as proportions using indicator specific denominators where required since the denominators varied in some indicators. The validity of an EFA was tested using Bartlett’s test and Kaiser-Meyer-Olkin (KMO) test. The former tests whether the correlation matrix is an identity matrix and the latter tests whether the partial correlation coefficients are small [22, 23]. The KMO and Bartlett’s tests show that the data is appropriate for an EFA, KMO = 0.72, and *P*-value < 0.01 respectively. Three factors were retained, accounting for 51.58, 15.14 and 14.26% of the variance respectively. The factor scores were obtained as a linear combination of the standardised indicators.

The principle factor method was used to extract the factors from a correlation matrix of which the diagonal component are estimated communalities instead of having a value of 1 [24]. The decision on the number of factors to retain was based on the Kaiser Rule and interpretability. The eigenvalue is the amount of common variance that is explained by each factor. Since the indicators are not assumed to be mutually exclusive to one factor, an oblique rotation was used to allow the resulting factors to correlate. This method allows the factor axes identified from the initial extraction to rotate to give a simple structure and hence the factors have become more interpretable [21]. Also, as each Governorate has a proportion of every indicator, therefore a variable loading on two factors is acceptable.

The resulting three factors have been used to calculate a standardised index (SI) of SES. An initial index had to be calculated first, which takes into account the eigenvalue of each factor as weights of each extracted factor in explaining the total variance:

1 $$ II=\left[\left({F}_1\right)\left({W}_1\right)\right]+\left[\left({F}_2\right)\left({W}_2\right)\right]+\left[\left({F}_3\right)\left({W}_3\right)\right] $$

where *F*_1_is the score of factor 1 and *W*_1_is the weight of factor 1. The values from this index give both positive and negative values, making it difficult to interpret the results. As an example, therefore, a SI based on the initial index has been calculated for the Abha Governorate as:

2 $$ {SI}_{abha}=\frac{II_{abha}-{\mathit{\operatorname{Min}}}_{II}}{{\mathit{\operatorname{Max}}}_{II}-{\mathit{\operatorname{Min}}}_{II}} $$

The SI index measures the SES of one Governorate relative to the difference between the most affluent (Max initial index) and least affluent Governorates (Min initial index). The results range from 100 to 0, where 100 is the score of the most affluent Governorate and 0 is the score of the most deprived Governorate. The analysis was performed in Stata software version 12 [25].

### Socioeconomic classes

The latent class analysis (LCA) approach was used to produce a categorical index of SES. The analysis fits a model that identifies a subset of latent classes to the data and then generates probabilities for each Governorate to belong to each class. Then Governorates are classified into classes by modal assignment, in which they belong to the class with the highest probability [26].

The maximum likelihood method was used to estimate the LCA model parameters. To avoid the problem of sample size related non-convergence (local maxima), the indicator variables used were limited to 11 which represent socioeconomic disadvantages which are “Not in the labour force”, “Illiterate”, “No car”, “Living in traditional housing”, “Living in one floor in a traditional house”, “House not owned”, “No phone”, “No television”, “No internet”, “No library” and “No satellite” (Table 1). This is to allow for the rule of thumb which states that a minimum of 10 observations are needed for each indicator variable [27]. Furthermore, to ensure a global maximum solution, the number of random starting values were increased to 1000, and 100 optimizations were used [28].

The process started by estimating a one class model and increasing the number of classes gradually to a k-class model (27). The model fit indices examined have included the Akaike Information Criterion (AIC), Bayes Information Criterion (BIC), the sample size-adjusted BIC, the Lo-Mendel-Rubin likelihood ratio test (LMR-LRT), the bootstrapped likelihood ratio difference test (BLRT) and the entropy (27). The lower the values of the AIC, BIC and the sample-size adjusted BIC, the better the model fit. A nonsignificant *p*-value (*P* < 0.05) of LMR-LRT and BLRT indicates that the k-1 model is the most parsimonious model. An entropy value close to one indicates a good classification (27). In any case, BIC and the BLRT statistics are recommended and are superior to other test statistics as evidenced by a recent simulation study [29].

In our data, the entropy is 0.98 for a 2-class and a 4-class solution as well as the *P*-value being significant (< 0.01) for both. However, a 4-class solution attained the lowest value of AIC, BIC, adjusted BIC, and BLRT, as well as yielding a classification that was clearly distinct and interpretable, and this was chosen for the LCA (Table 2). Analysis was performed in Mplus version 7 [28].

**Table 2:** Fit statistics for latent class analysis incorporating 11 indicator variables

	1 class	2 classes	3 classes	4 classes	5 classes^a^
Parameters	22	34	46	58	70
H0 value	977.32	1318.219	1415.645	1567.115	–
AIC	− 1910.64	− 2568.44	− 2739.29	− 3018.23	–
BIC	− 1849.69	− 2474.23	− 2611.84	− 2857.53	–
Adj BIC	− 1919.23	− 2581.72	− 2757.26	− 3040.88	–
Entropy	n/a	0.984	0.95	0.984	–
VLMR	n/a	0.0004	0.4163	0.0281	–
BLRT	n/a	0	0	0	–

^a^The number of free parameters is too high to come up with trustworthy standard errors

## Results

The descriptive statistics on all indicator variables are given in Table 3. The “other tenure” category shows a slight skewness (3.33), as it includes homes that are given by charity or temporarily by a relative and these are very rare cases. The “higher education”, “other type of housing” and “other tenure” variables all have a very high kurtosis (12.46, 15.51 and 15.91 respectively). This indicates that the number of people with higher education, people who live in other types of homes and on other type of tenures is small, where the maximum values of these variables are 0.01, 0.54, and 0.07 respectively.

**Table 3:** Major categories and sub-categories of socioeconomic indictors and their descriptive statistics

Variables^a^	Mean	SD	Skewness	Kurtosis	Min	Max
Educational status						
Illiterate & read/write	00.40	00.09	00.43	03.74	00.18	00.73
School degree	.49	00.06	− 00.56	04.68	00.22	00.66
Diploma & university	.09	00.03	00.35	03.69	00.01	00.20
Higher education	0	0	02.55	12.46	0	00.01
Employment status						
In the labour force	.48	00.07	00.32	02.64	00.31	00.66
Students	.17	00.03	−00.54	03.94	00.05	00.23
Housewives	00.26	00.05	00.51	03.22	00.16	00.41
Retired	00.03	00.01	00.11	02.62	0	00.06
Other employment	00.03	00.01	00.86	03.37	0	00.08
Type of housing						
Traditional house (TH)	00.44	00.22	00.39	01.96	00.03	00.89
Villa	00.20	00.13	00.20	01.70	00.01	00.51
Floor in TH or villa	00.07	00.05	00.78	02.74	0	00.23
Apartment	00.17	00.14	01.51	04.94	0	00.64
Other type of housing	00.10	00.07	02.73	15.51	00.01	00.54
Tenure of housing						
Home owned	00.57	00.14	−00.32	02.37	00.24	00.84
Home rented	00.26	00.12	00.79	03.53	0	00.63
Home provided	00.15	00.09	01.41	05.57	00.02	00.51
Other tenure	00.01	00.01	03.33	15.91	0	00.07
Car ownership						
No car available	00.29	00.09	00.78	03.00	00.12	00.53
One car	00.46	00.07	00.35	03.29	00.27	00.69
Two or more cars	00.24	00.08	−00.33	02.07	00.03	00.38
Household amenities						
Phone not available	00.46	00.19	00.66	02.66	00.13	00.96
TV not available	00.21	00.12	01.30	05.39	00.03	00.72
PC not available	00.80	00.11	−00.96	03.93	00.43	00.98
Internet not available	00.87	00.08	−01.36	05.16	00.58	00.99
Library not available	00.84	00.08	−00.70	03.15	00.57	00.98
Satellite not available	00.63	00.17	−00.57	03.14	00.13	00.97
Video not available	00.78	00.13	−00.85	− 03.25	00.35	00.98
Video games not available	00.77	00.09	−00.34	02.85	00.48	00.97

^a^Numbers are proportions, calculated by using indicator specific denominators

### Standardised index of SES

Table 4 shows the loadings of the three factors. The “Students” indicator variable was found to be highly loading on Factors 2 and 3 (0.65 and 0.62 respectively). Factor 1 described the Governorates with a large proportion of the population that are mostly educated, in the labour force, live in rented apartments, own a car and have all important household items. Factor 2 described Governorates with a large proportion of the population who live in villas and own two or more cars. Factor 3 described Governorates with a large proportion of the population who are not in the labour force, live in traditional houses that are owned not rented.

**Table 4:** Factor loadings from principle component factor analysis after oblique rotation

Variable	Factor1	Factor2	Factor3
Educational status			
Illiterate & read/write	−0.90		
School degree	0.83		
Diploma & university	0.80		
Higher education	0.81		
Employment status			
In the labour force	0.64		−0.66
Student		0.65	0.62
Housewives	−0.60		0.38
Retired	−0.60		
Other employment	−0.63		0.42
Type of housing			
Traditional house (TH)	−0.70		0.30
Villa		0.62	
A floor in a TH or villa	0.41	0.41	
Apartment	0.86	−0.36	
Other type of housing	−0.38		−0.81
Tenure of housing			
Home owned	−0.75		0.43
Home rented	0.89		
Home provided			−0.81
Other type of tenure		−0.32	0.30
Car ownership			
No car	−0.45	−0.44	
One car	0.48	−0.42	0.33
Household amenities			
Two or more cars		0.86	
Phone available	0.80		
TV available	0.75		
PC available	0.90		
Internet available	0.92		
Library available	0.90		
Satellite available	0.88		
Video available	0.86		
Video games available	0.91		

The proportions of the eigenvalues of the three factors have been used in calculating the initial index from eq. 1, which resulted in Governorates ranging from 37.83 for the most affluent Governorate, Qatif, to − 43.09 for the most deprived Governorate, Kharkheer. These were then standardised to range from 100 to 0 (Additional file 1).

### Socioeconomic classes

The model fit statistics for a 1 to 5-class solution are presented in Table 2. Class 1 has 13 Governorates (11.01%) and is labelled the “affluent class”, class 2 has 53 Governorates (44.91%) and is labelled the “upper middle class”, class 3 has 39 Governorates (33.05%) and is labelled the “lower middle class” and class 4 has 13 Governorates (11.01%) and is labelled “the deprived class” (Additional file 2).

**Fig. 1:**
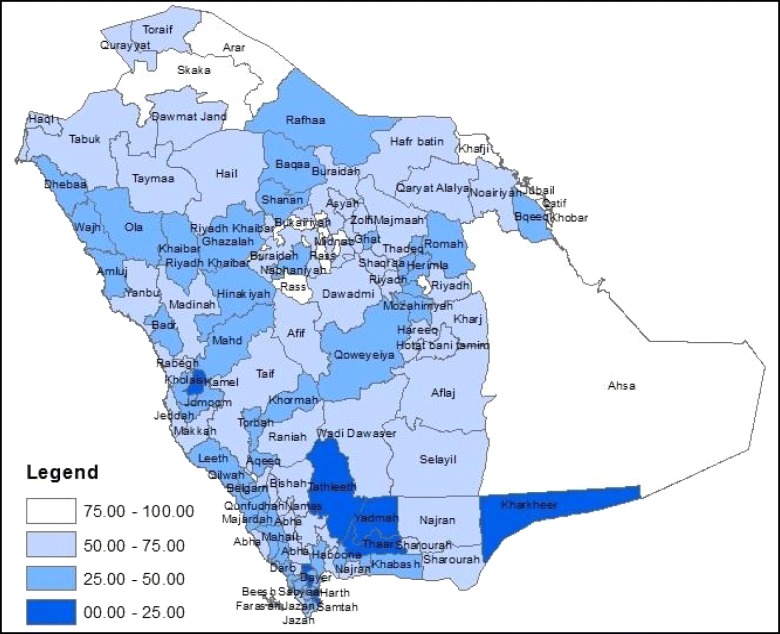
Map of Saudi Arabia illustrating the boundaries of the Governorates and their ranking from the most affluent to the most deprived as demonstrated by the standardised index of socioeconomic status. *The Empty Quarter desert is included in the border of the Ahsa Governorate, only 18% of this Governorate is inhabited

**Fig. 2:**
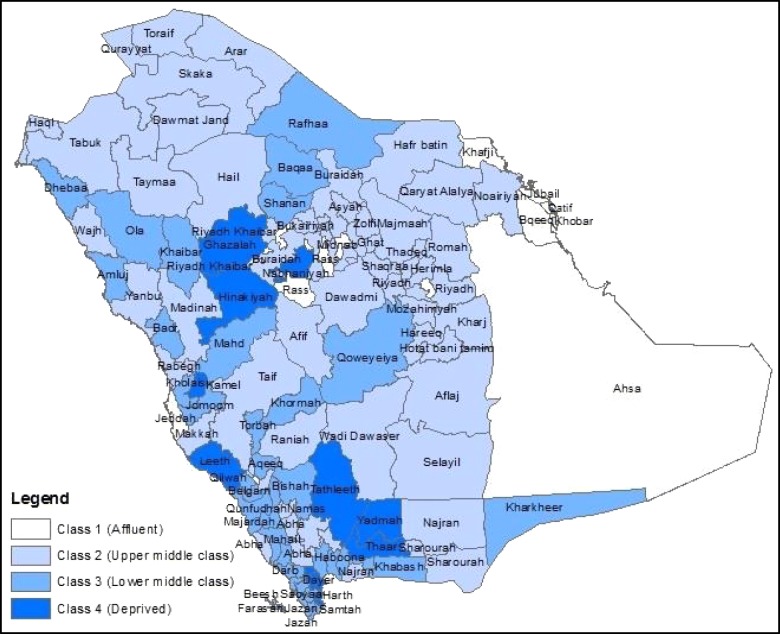
Map of Saudi Arabia illustrating the boundaries of the Governorates and their socioeconomic classes as demonstrated by the classes of socioeconomic status. *The Empty Quarter desert is included in the border of the Ahsa Governorate, only 18% of this Governorate is inhabited

Figures 1 and 2 illustrate how the Governorates are distributed geographically according to the standardised index and the SES class index.

The Figs. 1 and 2 show that the metropolitan Governorates of Riyadh, Jeddah and Dammam are affluent, as was expected. In the standardised index, the Kharkheer Governorate is the most deprived. Kharkheer is a very rural small Governorate and it is over 300 miles away from the closest area, Sharourah. One of the minor differences between the two indices is that Kharkheer is assigned to the “lower middle class” in the SES class index.

## Discussion

This is the first study that has attempted to develop area-based measures of SES in SA using routinely collected census data. Two replicable indices of SES have been successfully formulated using multiple socioeconomic indicators available from the 2004 national census data at the Governorate level. Previous studies of health in SA have not looked at SES in relation to their outcome of interest, due to the lack of any indices [30, 31].

Although the two indices have been formulated using two very different statistical methods, they have yielded very similar results, which indicates validity. The EFA method focuses on grouping variables, whereas the LCA method focuses on grouping observations. Therefore, small differences are to be expected. Nonetheless, the general pattern of affluency and deprivation of the Governorates were almost the same, only minor differences were found in the more deprived Governorates.

The two indices have shown that 8 out of 11 Governorates in the Eastern province are within the most affluent Governorates of the country (Qatif, Khobar, Rass Tanourah, Jubail, Dammam, Bqeeq, Ahsa and Khafji). The Eastern province hosts 5 major universities within the country, as well as ARAMCO, one of the world’s largest oil companies. The Jubail Governorate in the northern part of the Eastern region is the largest industrial city in the Middle East. Therefore, it is understandable to find these Governorates to be within the most affluent, especially that the Eastern province is the gate towards the rest of the gulf countries. The major cities in the country, such as the capital Riyadh and Jeddah were also within the affluent ranking in the SI and affluent ranking from the SES class index.

The selection of indicators was critical. The analysis began with a close examination of what has been chosen to formulate socioeconomic indices in other countries. The Townsend and Carstairs indices in the UK, as well as the indices in the US and Spain have all utilised indicators available from national censuses. Unlike the Saudi census, the censuses used in formulating these indices have included important indicators such as unemployment and overcrowding. Although the Saudi census does have a variable of employment status, this variable only shows the proportion that are in the labour force, and this variable includes the proportion of the population who are available for work, hence it includes both the employed and the unemployed. Furthermore, the Saudi census collects information on the number of rooms per household, therefore the information necessary to create an overcrowding variable are available but have not been accessible. Consequently, a decision was made that balanced between what had to be included and what was available and accessible from the census. Of interest is the student variable, which had a dichotomy on two factors, Factor 2 and Factor 3. A Governorate that has a high proportion of students may indicate that the Governorate is either affluent or poor. This variable covers the population aged > 15 years. Therefore, it includes both university students and mature students (> 21 years old) returning to schools to gain better jobs. Both of which explain why the variable loads highly onto the two factors.

Similar to the Townsend index [8], the final numerical value assigned to each Governorate in the standardised index does not measure a specific object. The value provides an abstract measure of deprivation that is used to rank these Governorates [32]. The standardised index method has been used to develop an index for Canada [33], India [34, 35], Japan [36] and the State of Mississippi in the USA [37]. However, none of these studies have attempted to re-construct the index using another statistical method for extra validity. The LCA approach was chosen to be able to construct a categorical class of SES to be used in future analyses; hence, Saudi researchers have the choice between a continuous and a categorical index.

Naturally, with the standardised index, more detail is given with regards to where a certain Governorate stands compared to the others, in contrast to the socioeconomic class index where Governorates are aggregated into four classes, each sharing similar characteristics amongst them. If we were to compare the results of these indices with those of Townsend and Carstairs - calculated on the census ward level - it can be seen that the rural wards had lower scores demonstrating affluence in these areas whilst the urban areas had higher scores demonstrating deprivation. In contrast, in Saudi Arabia, rural Governorates had lower scores in the standardised index and were categorised as either being in the ‘lower middle class’ or the ‘most deprived class’ in the class index. It is one of the societal characteristics of Saudi Arabia to consolidate in one specific area that has higher educational institutions and specialised healthcare services. Usually these are associated with more job opportunities for the unemployed and have more economical and financial opportunities for those interested in engaging in businesses. Therefore, these areas are generally characterised by affluence.

Although, the two indices have been able to pinpoint the most deprived Governorates, thus aiding in policy making, as well as their validity for use with data collected on a national scale, such as disease registries. They do have a few limitations. First, the indices are on a Governorate level, and Governorates range from large cities such as the capital Riyadh to small villages such as Kharkheer. Misclassification bias is a problem that occurs with categorical variables such as that of the socioeconomic class index, as a result of assigning each Governorate to a class, for example, assigning Riyadh to the affluent class, hence, assuming that all the population of Riyadh are affluent, which is not true.

The use of the 2004 census is arguably outdated and may not reflect the current situation within SA. However, considering that these socioeconomic indices have been developed for the first time, it is worth using the 2004 census which was carried out at a time prior to the social, cultural, economical and educational changes that have been implemented within the country. Such changes have included the establishment of over 26 universities across all provinces in SA, as well as the establishment of The Custodian of The Two Holy Mosques’ Overseas Scholarship Program in 2005 which has supported tens of thousands of Saudis’ to study in top ranking universities worldwide in diverse scientific programs [38]. This would allow us to compare and contrast the results of these indices with those that will be developed based on the 2010 census in a rapidly changing country.

The modifiable aerial unit problem is a methodological issue, whereby the choice of this geographical scale is made based on administrative/political convenience rather than being based on sound empirical evidence [39]. One way to overcome these problems is to be able to reproduce these indices on a finer geographical scale. The introduction of the new postal system that has started in 2011 is an excellent new opportunity and has motivated this work. It will facilitate the process of selecting smaller boundaries, for example a residential zip code to be able to assign individuals to spatial locations. Smaller boundaries will ensure that the population within these areas are more homogenous, hence, increasing the reliability of these analyses. Those health organisations wishing to use area-based measures of SES should incorporate these newly developed residential addresses into their databases for data linkage.

## Conclusions

The examination of area-based SES will encourage the study of health inequalities in SA, and provide a valid measure to be used in future researches, as well as provide disease registries in Saudi with the opportunity of including these measures within the patient information to facilitate their use among researchers. In conclusion, two replicable area-based measures of SES have been constructed for the 118 Governorates of SA. The indices have shown very similar results, mainly metropolitan cities are the most affluent groups, and the smaller villages to be within the most deprived. These indices may be updated and replicated with the publication of the next national census.

## Additional files

Additional file 1: Title and description of data: Results of the standardised index of socioeconomic status for the 118 Governorates of Saudi Arabia. (DOCX 25 kb)

Additional file 2: Title and description of data: Results of the index of socioeconomic classes for the 118 Governorates of Saudi Arabia. (DOCX 22 kb)

## Abbreviations

AIC: Akaika Information Criterion
BIC: Bayes Information Criterion
BLRT: Bootstrapped likelihood ratio difference test
EFA: Exploratory factor analysis
KMO: Kaiser-Meyer-Olkin
LCA: Latent class analysis
LMR-LRT: Lo-Mendel-Rubin likelihood ratio test
SA: Saudi Arabia
SES: Socioeconomic status
SI: Standardised index
