# Supplementary material for: Development of two socioeconomic indices for Saudi Arabia
Source: BMC Public Health. 2018 Jun 26;18:791. doi: 10.1186/s12889-018-5723-z (PMC6019717; doi:10.1186/s12889-018-5723-z)
Supplement: Supplementary file 1 — Title and description of data: Results of the standardised index of socioeconomic status for the 118 Governorates of Saudi Arabia. (DOCX 25 kb) [file 12889_2018_5723_MOESM1_ESM.docx]

Results of the standardised index of socioeconomic status for the 118 Governorates of Saudi Arabia

| **Province** | **Governorate** | **Initial index** | **Standardised index** |
| --- | --- | --- | --- |
| Eastern Region | Qatif | 131.25 | 100.00 |
| Eastern Region | Khobar | 122.70 | 97.02 |
| Eastern Region | Rass Tanourah | 113.03 | 93.64 |
| Eastern Region | Jubail | 106.89 | 91.50 |
| Riyadh | Riyadh | 104.71 | 90.74 |
| Eastern Region | Dammam | 104.08 | 90.52 |
| Eastern Region | Bqeeq | 89.92 | 85.59 |
| Eastern Region | Ahsa | 86.56 | 84.41 |
| Eastern Region | Khafji | 84.11 | 83.56 |
| Qassim | Onaizah | 82.63 | 83.04 |
| Baha | Baha | 80.88 | 82.43 |
| Northern | Arar | 72.58 | 79.53 |
| Jouf | Skaka | 62.74 | 76.10 |
| Riyadh | Dareiyah | 60.72 | 75.40 |
| Qassim | Buraidah | 62.27 | 75.94 |
| Jouf | Qurayaat | 57.65 | 74.33 |
| Makkah | Jeddah | 57.83 | 74.39 |
| Hail | Hail | 58.07 | 74.48 |
| Madinah | Madinah | 56.78 | 74.02 |
| Tabuk | Tabuk | 57.32 | 74.21 |
| Qassim | Rass | 55.87 | 73.71 |
| Aseer | Abha | 55.92 | 73.72 |
| Jouf | Dawmat Jand | 53.19 | 72.77 |
| Qassim | Badae | 50.97 | 72.00 |
| Aseer | Khamis Mushayt | 48.86 | 71.26 |
| Madinah | Yanbu | 47.68 | 70.85 |
| Northern | Toraif | 45.62 | 70.13 |
| Riyadh | Zolfi | 42.84 | 69.16 |
| Baha | Mandaq | 40.32 | 68.28 |
| Riyadh | Shaqraa | 41.28 | 68.62 |
| Makkah | Taif | 38.50 | 67.65 |
| Eastern Region | Hafr batin | 38.80 | 67.76 |
| Riyadh | Kharj | 37.91 | 67.44 |
| Baha | Baljurashi | 36.02 | 66.78 |
| Makkah | Makkah | 36.65 | 67.01 |
| Northern | Rafha | 29.70 | 64.58 |
| Qassim | Asyah | 30.24 | 64.77 |
| Qassim | Midnab | 28.53 | 64.17 |
| Riyadh | Majmaah | 27.64 | 63.86 |
| Qassim | Shamssiyah | 26.13 | 63.33 |
| Aseer | Namas | 20.62 | 61.41 |
| Qassim | Bikairiah | 20.17 | 61.26 |
| Riyadh | Hotat bani tamim | 19.64 | 61.07 |
| Tabuk | Haql | 21.86 | 61.84 |
| Qassim | Riaydh Khobaraa | 18.08 | 60.53 |
| Aseer | Uhd Rofidah | 17.03 | 60.16 |
| Najran | Najran | 13.95 | 59.09 |
| Najran | Sharourah | 10.69 | 57.95 |
| Riyadh | Selayil | 11.77 | 58.33 |
| Baha | Quraa | 6.25 | 56.40 |
| Eastern Region | Noariyah | 8.15 | 57.06 |
| Jazan | Jazan | 9.25 | 57.45 |
| Riyadh | Afeef | 11.28 | 58.15 |
| Eastern Region | Qaryat Alya | 3.06 | 55.29 |
| Aseer | Belgarn | 6.47 | 56.48 |
| Riyadh | Dawadmi | 4.79 | 55.89 |
| Qassim | Oyoon Jawaa | 4.80 | 55.90 |
| Riyadh | Hareeq | -1.41 | 53.73 |
| Riyadh | Aflaj | 0.11 | 54.26 |
| Jazan | Farasan | -2.01 | 53.52 |
| Aseer | Dhahran Janoub | -2.21 | 53.45 |
| Riyadh | Wadi Dawaser | -4.86 | 52.53 |
| Tabuk | Tayma | -5.26 | 52.39 |
| Makkah | Rabegh | -8.69 | 51.19 |
| Aseer | Bishah | -11.21 | 50.31 |
| Riyadh | Ghat | -8.80 | 51.15 |
| Madinah | Badr | -12.09 | 50.00 |
| Tabuk | Wajh | -12.38 | 49.90 |
| Jazan | Abu Arish | -15.04 | 48.98 |
| Baha | Mikhwah | -16.21 | 48.57 |
| Tabuk | Amluj | -16.22 | 48.56 |
| Makkah | Kholais | -18.29 | 47.84 |
| Aseer | Sarat Abaida | -18.36 | 47.82 |
| Riyadh | Romah | -24.33 | 45.73 |
| Makkah | Jomoom | -26.44 | 45.00 |
| Tabuk | Dhebaa | -25.71 | 45.25 |
| Makkah | Khormah | -27.93 | 44.48 |
| Jazan | Uhd Masarha | -31.48 | 43.24 |
| Riyadh | Thadeq | -28.73 | 44.20 |
| Aseer | Rejal Almaa | -31.72 | 43.16 |
| Makkah | Raniah | -34.29 | 42.26 |
| Aseer | Mahail | -32.13 | 43.02 |
| Jazan | Beesh | -34.73 | 42.11 |
| Jazan | Dhamd | -32.40 | 42.92 |
| Baha | Qilwah | -34.97 | 42.03 |
| Jazan | Sabyaa | -33.87 | 42.41 |
| Makkah | Qunfudhah | -35.41 | 41.87 |
| Madinah | Khaibar | -35.45 | 41.86 |
| Madinah | Ola | -35.41 | 41.87 |
| Hail | Baqaa | -39.79 | 40.34 |
| Aseer | Majardah | -40.53 | 40.08 |
| Riyadh | Mozahimyah | -40.18 | 40.21 |
| Najran | Haboona | -46.51 | 38.00 |
| Baha | Aqeeq | -44.39 | 38.74 |
| Makkah | Torbah | -43.16 | 39.17 |
| Jazan | Samtah | -45.51 | 38.35 |
| Riyadh | Herimla | -46.16 | 38.12 |
| Riyadh | Qowaiyah | -48.83 | 37.19 |
| Hail | Shanan | -51.53 | 36.25 |
| Najran | Badr Janoub | -56.75 | 34.43 |
| Madinah | Hinakiyah | -54.67 | 35.15 |
| Jazan | Darb | -56.06 | 34.67 |
| Makkah | Leeth | -55.07 | 35.01 |
| Qassim | Nabhanyah | -59.14 | 33.59 |
| Hail | Ghazalah | -62.56 | 32.40 |
| Madinah | Mahd | -66.19 | 31.13 |
| Riyadh | Dhurma | -68.99 | 30.16 |
| Najran | Khabash | -76.20 | 27.64 |
| Jazan | Dayer | -77.09 | 27.33 |
| Najran | Thaar | -85.75 | 24.31 |
| Aseer | Tathleeth | -88.30 | 23.42 |
| Makkah | Kamel | -90.79 | 22.55 |
| Jazan | Harth | -103.39 | 18.16 |
| Jazan | Aridhah | -103.08 | 18.27 |
| Jazan | Eidabi | -103.60 | 18.09 |
| Najran | Yadmah | -101.28 | 18.90 |
| Jazan | Reeth | -104.40 | 17.81 |
| Najran | Kharkheer | -155.46 | 0.00 |
