# Supplementary material for: Development of two socioeconomic indices for Saudi Arabia
Source: BMC Public Health. 2018 Jun 26;18:791. doi: 10.1186/s12889-018-5723-z (PMC6019717; doi:10.1186/s12889-018-5723-z)
Supplement: Supplementary file 2 — Title and description of data: Results of the index of socioeconomic classes for the 118 Governorates of Saudi Arabia. (DOCX 22 kb) [file 12889_2018_5723_MOESM2_ESM.docx]

Results of the index of socioeconomic classes for the 118 Governorates of Saudi Arabia

| **Province** | **Governorate** | **Class** |
| --- | --- | --- |
| Eastern region | Khobar | 1 |
| Eastern region | Jubail | 1 |
| Eastern region | Dammam | 1 |
| Riyadh | Riyadh | 1 |
| Eastern region | Rass Tanourah | 1 |
| Eastern region | Qatif | 1 |
| Makkah | Jeddah | 1 |
| Qassim | Onaizah | 1 |
| Eastern region | Bqeeq | 1 |
| Riyadh | Dareiyah | 1 |
| Eastern region | Khafji | 1 |
| Eastern region | Ahsa | 1 |
| Baha | Baha | 1 |
| Tabuk | Tabuk | 2 |
| Madinah | Madinah | 2 |
| Madinah | Yanbu | 2 |
| Northern | Arar | 2 |
| Riyadh | Kharj | 2 |
| Qassim | Buraidah | 2 |
| Riyadh | Shaqraa | 2 |
| Aseer | Khamis Mushayt | 2 |
| Aseer | Abha | 2 |
| Qassim | Rass | 2 |
| Makkah | Makkah | 2 |
| Jouf | Skaka | 2 |
| Qassim | Badae | 2 |
| Eastern region | Hafr batin | 2 |
| Hail | Hail | 2 |
| Jouf | Dawmat Jand | 2 |
| Northern | Toraif | 2 |
| Riyadh | Zolfi | 2 |
| Makkah | Taif | 2 |
| Jouf | Qurayaat | 2 |
| Qassim | Midnab | 2 |
| Riyadh | Majmaah | 2 |
| Baha | Baljurashi | 2 |
| Najran | Sharourah | 2 |
| Riyadh | Hareeq | 2 |
| Riyadh | Selayil | 2 |
| Riyadh | Hotat bani tamim | 2 |
| Tabuk | Haql | 2 |
| Qassim | Bikairiah | 2 |
| Najran | Najran | 2 |
| Qassim | Asyah | 2 |
| Riyadh | Ghat | 2 |
| Northern | Rafha | 2 |
| Baha | Mandaq | 2 |
| Qassim | Oyoon Jawaa | 2 |
| Aseer | Uhd Rofidah | 2 |
| Aseer | Namas | 2 |
| Qassim | Shamssiyah | 2 |
| Eastern region | Noariyah | 2 |
| Riyadh | Herimla | 2 |
| Qassim | Riaydh Khobaraa | 2 |
| Riyadh | Aflaj | 2 |
| Riyadh | Mozahimyah | 2 |
| Riyadh | Wadi Dawaser | 2 |
| Eastern region | Qaryat Alya | 2 |
| Riyadh | Romah | 2 |
| Riyadh | Dawadmi | 2 |
| Riyadh | Afeef | 2 |
| Tabuk | Wajh | 2 |
| Tabuk | Tayma | 2 |
| Baha | Quraa | 2 |
| Riyadh | Thadeq | 2 |
| Riyadh | Dhurma | 2 |
| Jazan | Jazan | 3 |
| Makkah | Rabegh | 3 |
| Aseer | Belgarn | 3 |
| Tabuk | Dhebaa | 3 |
| Tabuk | Amluj | 3 |
| Jazan | Farasan | 3 |
| Aseer | Bishah | 3 |
| Aseer | Dhahran Janoub | 3 |
| Madinah | Ola | 3 |
| Madinah | Badr | 3 |
| Makkah | Khormah | 3 |
| Jazan | Abu Arish | 3 |
| Makkah | Raniah | 3 |
| Hail | Baqaa | 3 |
| Riyadh | Qowaiyah | 3 |
| Makkah | Kholais | 3 |
| Baha | Mikhwah | 3 |
| Aseer | Sarat Abaida | 3 |
| Makkah | Jomoom | 3 |
| Najran | Haboona | 3 |
| Makkah | Torbah | 3 |
| Hail | Shanan | 3 |
| Aseer | Rejal Almaa | 3 |
| Jazan | Sabyaa | 3 |
| Madinah | Khaibar | 3 |
| Baha | Aqeeq | 3 |
| Aseer | Mahail | 3 |
| Jazan | Beesh | 3 |
| Jazan | Uhd Masarha | 3 |
| Jazan | Dhamd | 3 |
| Makkah | Qunfudhah | 3 |
| Aseer | Majardah | 3 |
| Baha | Qilwah | 3 |
| Jazan | Darb | 3 |
| Najran | Badr Janoub | 3 |
| Madinah | Mahd | 3 |
| Jazan | Samtah | 3 |
| Najran | Khabash | 3 |
| Najran | Kharkheer | 3 |
| Madinah | Hinakiyah | 4 |
| Qassim | Nabhanyah | 4 |
| Makkah | Leeth | 4 |
| Hail | Ghazalah | 4 |
| Aseer | Tathleeth | 4 |
| Jazan | Dayer | 4 |
| Najran | Thaar | 4 |
| Makkah | Kamel | 4 |
| Najran | Yadmah | 4 |
| Jazan | Harth | 4 |
| Jazan | Eidabi | 4 |
| Jazan | Aridhah | 4 |
| Jazan | Reeth | 4 |
